# Gentamicin nephrotoxicity in animals: Current knowledge and future perspectives

**DOI:** 10.17179/excli2017-165

**Published:** 2017-03-24

**Authors:** Pavle Randjelovic, Slavimir Veljkovic, Nenad Stojiljkovic, Dušan Sokolovic, Ivan Ilic

**Affiliations:** 1 University of Niš, Faculty of Medicine, Department of Physiology, Niš, Serbia; 2 University of Niš, Faculty of Medicine, Department of Biochemistry, Niš, Serbia; 3 University of Niš, Faculty of Medicine, Institute of Pathology, Niš, Serbia

**Keywords:** gentamicin, kidney, nephrotoxicity, aminoglycoside, oxidative stress, proliferation

## Abstract

Due to high relative blood flow the kidney is prone to drug-induced damage. Aminoglycoside type antibiotic gentamicin is one of the leading cause of drug-induced nephrotoxicity. In recent years gentamicin nephrotoxicity is significantly reduced by shifting to once daily dosage as well as by eliminating known risk factors. Application of gentamicin is still related to serious side effects which are reported more often compared to other antibiotics. Because gentamicin is still heavily used and is highly efficient in treating infections, it is important to find mechanisms to reduce its nephrotoxicity. This aim can only be achieved through better understanding of kidney metabolism of gentamicin. This problem has been extensively researched in the last 20 years. The experimental results have provided evidence for almost complete understanding of mechanisms responsible for gentamicin nephrotoxicity. We now have well described morphological, biochemical and functional changes in kidney due to gentamicin application. During the years, this model has become so popular that now it is used as an experimental model for nephrotoxicity per se. This situation can mislead an ordinary reader of scientific literature that we know everything about it and there is nothing new to discover here. But quite opposite is true. The precise and complete mechanism of gentamicin nephrotoxicity is still point of speculation and an unfinished story. With emerge of new and versatile technics in biomedicine we have an opportunity to reexamine old beliefs and discover new facts. This review focuses on current knowledge in this area and gives some future perspectives.

## Introduction

Due to relatively large blood flow (20 % of stroke volume) and the ability to extract and concentrate hydrosoluble toxic molecules, the kidney is prone to drug induced damage. The experimental data point to the fact that drug induced nephrotoxicity includes multiple mechanisms that can be classified as vascular, glomerular and tubular. The kidney damage is usually a consequence of tubular obsruction caused by cell swelling or debris deposition (Perazella, 2003[61]). Toxic substances can damage various cell types in kidney. The most studied effect is necrosis of tubular epithelial cells. There is also a description of morphological glomerular damage with reduced function (Stojiljkovic et al., 2012[85]). The application of some drugs (gentamicin, cisplatin) to experimental animals is causing significant reduction in kidney blood flow and glomerular filtration with increase in vascular resistance. These effects can be present without permanent morphological changes in glomerulus and independent from tubular damage (Bennett et al., 1994[8]). 

## Tubular Effects

Gentamicin (GM) is causing tubular damage through: 1) necrosis of tubular epithelial cells, predominantly in proximal segment and 2) alteration of function of main cellular components involved in transport of water and solutes. The central aspect of GM nephrotoxicity is tubular cytotoxicity. The treatment of animals with GM is related to apoptosis (El Mouedden et al., 2000[22]; Li et al., 2009[42]) as well as necrosis of tubular epithelial cells (Edwards et al., 2007[21]). In cell culture, GM is also causing apoptosis (El Mouedden et al., 2000[22]) as well as necrosis of those cells (Pessoa et al., 2009[62]). Gentamicin is causing cytotoxicity in the cells where it accumulates. In kidney, those are usually epithelial cells of proximal tubules (Verpooten et al., 1989[91]) while cells of distal tubules and collecting ducts are significantly less affected by cytotoxic effects (Fujiwara et al., 2009[24]). The increased accumulation of GM in proximal tubules is related to expression of transport molecule for proteins and cations (megalin and cubilin complex) in proximal tubules. It is known that this complex is responsible for transport of GM by endocytosis (Sassen et al., 2006[77]). The drug is then transported to lysosomes, Goldzi apparatus and endoplasmic reticulum (Silverblatt, 1982[82]). Gentamicin binds to membrane phospholipids, alters its function and lead to a condition known as phospholipidosis in humans (De Broe et al., 1984[16]) and experimental animals (Nonclercq et al., 1992[58]). Lysosomal phospholipidosis is caused by: disorder in phosphatidylinositol signaling pathway (Ramsammy et al., 1988[65]), reduced turnover of phospholipids and their accumulation in plasma membrane (Laurent et al., 1982[40]), decrease in the available negative charge for proper function of phospholipases (Mingeot-Leclercq et al., 1995[50]), inhibition of calcium dependent phosphodiesterases (Van Rooijen and Agranoff, 1985[90]) and by inhibition of phospholipases A1, A2 and C1 (Abdel-Gayoum et al., 1993[1]). Phospholipidosis is directly related to toxicity of GM (Kaloyanides, 1992[33]). However, similar changes were detected in other cell types exposed to GM but without significant damage and cellular death. We can conclude that these effects are not important to toxicity in epithelial cells. When concentration of GM in endosomes reaches certain level, their membranes disrupts and their content including GM is released to cytoplasm (Regec et al., 1989[69]). In cytoplasm GM acts on mitochondria both directly and indirectly (Morales et al., 2010[53]) and activates intrinsic apoptosis pathway, breaks respiratory chain, decrease ATP synthesis and leads to oxidative stress by creating superoxide anion and hydroxyl radical (Cuzzocrea et al., 2002[15]) resulting in cell death. Indirect mitochondrial effect is mediated by increased levels of Bcl-2 associated protein X (Bax) (Horibe et al., 2004[28]) through inhibition of its degradation in proteasomes (Servais et al., 2006[80]). Lysosomal content is made of highly active proteases called cathepsins capable of inducing cell death (Schnellmann and Williams, 1998[78]). Cathepsin induced cell death is performed through apoptosis mechanism by direct activation of executive caspases and indirectly by proteolitic activation of proapoptotic protein Bid and intrinsic pathway (Yin, 2006[93]). When present in large quantities, cathepsins cause massive proteolysis which results in necrosis, especially in the lack of ATP (Golstein and Kroemer, 2007[25]). Inside endoplasmic reticulum, GM inhibits protein synthesis, disrupts translation accuracy and posttranslation modification of proteins (Horibe et al., 2004[28]). This results in endoplasmic reticulum stress and activation of apoptosis by caspase 12 and calpain (Peyrou et al., 2007[63]). Finally, it is documented that activation of extracellular calcium receptor (CaSR) by GM also induce milder degree of apoptosis in cells of tubules with CaSR but also in those who lacks it. On the other hand, CaSR is found on GM resistant cells like bone cells, neurons, smooth muscle cells, endothelial cells etc. So we still need more information about the precise role of CaSR in GM-induced nephrotoxicity.

In cell culture experiments it was shown da GM inhibits a number of membrane transport proteins, independently of cell damage (Sassen et al., 2006[77]). Inhibition of transport disrupts reabsorption in tubules and threatens cell homeostasis. For example, sodium-potassium pump is key component in regulation of cell volume. Inhibition of this pump would produce cell swelling and consequential necrosis or apoptosis (Lieberthal and Levine, 1996[43]). As soon as after 30 minutes of perfusion of kidney with GM or 3 hours after application to animals, reduced kidney calcium and magnesium uptake is seen, leading to increased excretion of this minerals through kidney, before visible signs of damage (Parsons et al., 1997[59]). Gentamicin is transported and competes with proteins, cations and other molecules for megalin-cubilin complex in proximal tubules and interferes with their reabsorption.

The damaged tissue or cells is released into the lumen of the tubules and leads to partial or complete obstruction resulting in decrease of excretory function (Rivas-Cabanero et al., 1993[70]). The present obstruction increases the hydrostatic pressure in tubules and Bowman's capsule which leads to decrease in filtration pressure and glomerular filtration. However, in the beginning of this process, when there is no significant tubular obstruction, we can detect a rise in serum levels of urea and creatinine, pointing to reduction in glomerular filtration. In the absence of marked obstruction of the nephron, the observed increase in plasma creatinine level can only be explained by reduction in glomerular filtration. Structural damage of the tubules leads to impairment of reabsorption causing overflow of water and solutes into the distal parts of nephron, activating tubuloglomerular feedback loop. This physiological mechanism is accomplished by angiotensin II and afferent and efferent arteriole, resulting in decrease in glomerular filtration (Blantz et al., 2007[10]). This feedback is activated as a protective mechanism to prevent great loss of water and solutes (Komlosi et al., 2009[36]). This mechanism is fully adapted in 24 hours period (Deng et al., 2004[18]). So, after that time its role in filtration decrease should be absent in physiological conditions. Nevertheless, the filtration in glomerulus remains low as long as GM is present. One of the possible explanations is that present oxidative stress, inflammation and release of vasoconstrictor substances give rise to constriction of mesangial cells and blood vessels. 

There are two main factors for kidney damage induced by GM: its accumulation in proximal tubular cells and interaction with cell membranes and organelles. The cells of brush border are exposed to concentration of GM higher than the one in serum because kidney is main excretory organ for GM. There is evidence that the concentration of GM in cells of proximal tubules is not related to nephrotoxicity, although tubular necrosis is dose dependent (Bennett, 1989[7]). Recent *in vitro* studies showed that the key aspect of GM cytotoxicity is its concentration in cytoplasm, not accumulation in lysosomes as thought earlier (Servais et al., 2008[81]). Also it was proved that small amount of GM directly enters the cytoplasm, independently of megalin-cubilin mediated endocytosis (Myrdal et al., 2005[56]). Besides, it was demonstrated that GM can enter the cells of tubules *in vitro* through nonspecific cationic channel TRPV4 (Karasawa et al., 2008[34]). However, this channel is present only in distal tubules and its contribution to GM cell entry is small.

Gentamicin is actively eliminated by glomerular filtration. Around 3 to 5 % from entered GM is actively reabsorbed in proximal tubules cells (Laurent et al., 1990[39]) and cause necrosis of S1-S2 segment of proximal tubules (Houghton et al., 1976[29]). The accumulation of undigested phospholipids in lysosomes is tightly related to GM nephrotoxicity. The concentration of GM in lysosomes can be shown by autoradiography (Silverblatt and Kuehn, 1979[83]) or by labeled gold (Beauchamp et al., 1991[5]). In both humans and animals, lysosomal phospholipidosis results in cell necrosis and alterations in epithelial cells of proximal tubules and to a lesser extent in distal tubules and collecting ducts. Cojocel et al. (1984[13]) has shown that GM decrease level of glomerular filtration, as well as tubular reabsorption, leading to accumulation of low molecular weight proteins along with increased renal excretion of sodium and potassium. In physiological conditions these proteins are freely filtered in glomerulus and completely reabsorbed in tubules. Acidic environment enhance electrostatic interactions between GM and negatively charged phospholipids. Gentamicin easily binds to negatively charged lysosomal phospholipids because of highly acidic environment. The presence of this negatively charged phospholipids stimulates degradation of phosphatidylcholine by acid sphingomyelinase, phospholypase A1, A2 and lipophospholypase (Laurent et al., 1990[39]). However, it is not clear is the catabolism of other lipids altered in similar manner. The intensity of phospholipids degradation is reduced in proximal tubules in the presence of GM (Laurent et al., 1990[39]). This inhibitory effect of GM on phospholipases is being accompanied with the presence of myeloid bodies (Laurent et al., 1982[40]). In experimental animals and humans too the noted increase of phospholipids in kidney cortex and phospholipiduria are related to lysosomal damage (Ibrahim et al., 1989[30]). The accumulated phospholipids in lysosomes disturb the homeostasis of the cell and initiates burst of organelles with release of toxic components including GM. It was shown that poly-L-aspartic acid (PAA) can prevent lysosomal phospholipidosis caused by GM as well as signs of nephrotoxicity (Beauchamp et al., 1990[6]). The nephrotoxicity is reversible process. Stopping of GM treatment is followed with regeneration of kidney epithelia. The glomerular filtration is changed only when over 30 % of proximal tubules are necrotic (Kourilsky et al., 1982[38]). The reduced kidney function is seen when regeneration process can not compensate tissue damage cause by toxic agent. 

## Glomerular Effects

The glomerulus is the first part of nephron that comes in contact with GM. Multiple mechanisms are responsible for filtration changes. Gentamicin is causing mesangial contraction and reducing glomerular filtration (Martinez-Salgado et al., 2007[46]). Gentamicin also stimulates proliferation of mesangial cells followed by compensatory increase in apoptosis of those cells (Martinez-Salgado et al., 2007[46]). Although GM does not cause morphological changes in glomerulus, it was shown that high doses of GM are causing mild enlargement of glomeruli, change in circular shape and density with neutrophil infiltration (Stojiljkovic et al., 2008[84]). The loss of selectivity of filtration membrane leads to proteinuria, especially in situation of reduced reabsorption as seen in tubular necrosis (De-Barros-e-Silva et al., 1992[17]). Earlier studies showed that GM decrease number and size of pores on endothelial cells in glomerulus causing reduction in filtration (Cojocel et al., 1984[14]). Those effects are a consequence of mesangial contraction. Gentamicin activates contraction of mesangial cells in culture and decrease filtration (Martinez-Salgado et al., 1997[49]). The mesangial contraction could be mediated by several mechanisms: secretion of platelet activating factor (PAF) (Rodriguez-Barbero et al., 1995[73]), activation of renin-angiotensin system, production of vasoconstrictor substances like endothelin-1 and thromboxane A2 (Valdivielso et al., 1999[89]), stimulation of CaSR and increase in reactive oxygen species (ROS) and oxidative stress (Duque et al., 1992[20]). The activation of phospholipase A2 is related to synthesis of some of the mentioned mediators and with GM effect on mesangial cells (Martinez-Salgado et al., 1997[49]). Phospholipase A2 catalyze production of arachidonic acid and thromboxane A2 leading to mesangial contraction. Platelet activating factor is important mediator of mesangial contraction (Santos et al., 2009[76]). Blocking the effect of PAF results in lack of mesangial contraction and decrease in filtration caused by GM (Rodriguez-Barbero et al., 1997[72]). It seems that *in vitro* GM-induced cell proliferation is cell-specific. Some authors described that GM in *in vitro* conditions decrease proliferation of human epidermal keratinocyte, probably through inhibition of transport RNA (Tekos et al., 2003[86]). Also GM inhibits cell proliferation in the organ of Corti and cells similar to human osteoblasts (Bertolaso et al., 2003[9]). The observed differences in GM effects on various cell is probably dependent on drug concentration as well as experimental conditions. 

The experimental data are showing that O^2-^ is involved in apoptosis of mesangial cells caused by GM, because system xanthine and xanthine oxidase increase apoptosis similar to GM. Moreover, scavengers of ROS, superoxide dismutase (SOD) and catalase, inhibits GM induced apoptosis (Martinez-Salgado et al., 2004[44]). A lot of evidence points to the fact that stimulation of oxidative stress leads to apoptotic response in mesangial cells (Ishikawa and Kitamura, 2000[31]). Apoptosis of glomerular and tubular cells caused by interleukin 1α and tumor necrosis factor α (TNF-α) is mediated by ROS (Bohler et al., 2000[11]). Regarding this, O^2-^ is proposed as selective mediator of mesangial cell apoptosis caused by TNF-α (Moreno-Manzano et al., 2000[55]). Apoptosis of mesangial cells induced by GM is also characterized by early increase in proapoptotic protein Bax and late increase in antiapoptotic Bcl-2 protein (Martınez-Salgado et al., 2004[44]). Upregulation of Bcl-2 expression is also noted in cells of ovaries in response to geneticin (Tey et al., 2000[87]). Bcl-2 is found at sites of ROS production and is responsible for prevention of oxidative damage. It was shown that overexpression of Bcl-2 has protective effect on oxidative stress and apoptosis in neurons and mesangial cells (Sandau and Brüne, 2000[75]). The other function of Bcl-2 is antioxidative and prevention of apoptosis (Korsmeyer et al., 1995[37]). One can conclude that increased ratio of Bax/Bcl-2 has important role in GM induced apoptosis of mesangial cells.

Nitric oxide (NO) has vital function in local regulation of blood flow in kidney cortex and can influence glomerular filtration. NO can act in different ways, acting as physiological signal molecule, protector of cell functions and toxic mediator (Valdivielso and Blantz, 2002[88]). Cell damage mediated by NO is done through several mechanisms including disturbance of mitochondrial respiration, inhibition of enzymes, nitrosylation of proteins and lipid peroxidation by mediators like peroxynitrite produced in reaction between NO and O^2-^. This reaction happens when high concentration of NO become available as a result of increased activity of inducible NO synthase (iNOS) (Gordge, 1998[26]). We can assume that increased expression of iNOS and production of NO has a role in apoptosis of mesangial cells *in vitro* (Martinez-Salgado et al., 2004[44]). So, *in vitro* studies have shown that GM increase synthesis of iNOS messenger RNA in mesangial cells together with production of NO, and that NO has antiproliferative role in these cells treated with GM (Rivas-Cabanero et al., 1997[71]). In apoptosis of mesangial cells mediated by high intracellular calcium, NO acts as proapoptotic factor (Rodríguez-López et al., 1999[74]). Brüne (2002[12]) has reported that proapoptotic effect of NO on mesangial cells is partially mediated by activation of Jun N-terminal kinase (JNK). Even though the increased production of NO has more effect on apoptosis then on proliferation, the created O^2-^ as a result of stimulation of NO synthase could lead to proliferation of mesangial cells (Martinez-Salgado et al., 2002[45]). One should have in mind that both contraction and proliferation of mesangial cells in culture after treatment with GM are happening in artificial conditions without many *in vivo* neuroendocrine stimuli and cell-matrix interactions. That is why interpretation of *in vitro* results should be taken with caution. Generally, stimuli that cause *in vitro* contraction have similar effects *in vivo*. However, the proliferation process is more complex. Many stimuli are capable of inducting proliferation, apoptosis or other effects depending of specific conditions of the cell. 

There are numerous data about ability of calcium channel blockers to inhibit contraction and proliferation of mesangial cells induced by different agents (Montero et al., 1995[51]). Increase in free cytosolic calcium (Ca^2+^) is necessary for both processes in those cells. GM increase intracellular Ca^2+^ by stimulating entry of extracellular calcium and through releasing of internal depots (Martinez-Salgado et al., 2000[48]). Calcium channel blocker verapamil decrease contraction and proliferation of mesangial cells induced by GM, which proves that increase in intracellular calcium has important role in contractile and proliferative effects of GM (Martinez-Salgado et al., 2000[48]). During development of GM nephrotoxicity, the increased calcium concentration activates phospholipases, nucleases and proteases which disrupts the function of cell membrane and contribute to further damage. The protective effect of verapamil is based on calcium entry inhibition. Verapamil reduce liberation of calcium from endoplasmic reticulum. This is how verapamil reduce GM nephrotoxicity, which has redistribution of intracellular calcium in its core (Stojiljkovic et al., 2008[84]). 

The role of ROS in tubular and glomerular effects of GM has been clearly demonstrated. *In vivo*, ROS are labeled as proximal tubule necrosis and acute kidney injury mediators (Du and Yang, 1994[19]). Scavengers of ROS were found beneficial in attenuating kidney injury after application of GM (Randjelovic et al., 2012[67]). The application of antioxidants like SOD or dimethyl-thiourea prevented GM induced decrease of glomerular filtration (Nakajima et al., 1994[57]). The application of SOD in rats treated with GM leads to increased renal blood flow. This is why is considered that superoxide anion has some function in GM induced vasoconstriction (Nakajima et al., 1994[57]).

The role of ROS as physiological second messengers or inducers of oxidative stress is clearly evident in activation and injury of mesangial cells. There are reports that H_2_O_2_ is capable of inducing contraction of mesangial cells *in vitro*, as well as increase in preglomerular resistance *in vivo* (Duque et al., 1992[20]). Involvement of ROS in mesangial contraction and proliferation was demonstrated *in vivo* too. The application of GM leads to stimulation of O^2-^ and SOD activity. Also it was shown that intracellular ROS scavengers, SOD and catalase, inhibits GM induced mesangial proliferation and contraction (Martinez-Salgado et al., 2002[45]). Similar trend was shown for natural antioxidant trans-resveratrol (Morales et al., 2005[54]). The production of O^2-^ caused by GM in this cells is partly mediated by NADP(H) oxidase activity (Martinez-Salgado et al., 2002[45]). The other possible source of O^2-^ could be activation of phospholipase A2 (Pfeilschifter and Huwiler, 1994[64]). This is important because GM can activate phospholipase A2 (Martinez-Salgado et al., 1997[49]). There are numerous experimental data pointing that ROS can activate a mechanism leading to proliferation of mesangial cells (Ali, 1995[3]). Studies have confirmed that oxidative stress caused by application of xanthine with xanthine oxidase stimulates apoptosis of mesangial cells in culture, while addition of SOD and catalase in the medium prevents this effect (Martinez-Salgado et al., 2004[44]). 

In rats treated with GM the proliferation and apoptosis process are happening at the same time. Obviously there is mutual compensation, because there was not shown any change in the number of cells in mesangium (Martinez-Salgado et al., 2004[44]). There were no studies showing a change in the number of mesangial cells after GM treatment till 2013, when first data become available about the GM-induced increase in mesangial cell number (Randjelovic, 2013[66]). Our group of authors has shown that application of GM to rats is followed by increase in the size of glomeruli without the change in cellular density. A conclusion from such data was that there was an increase in cell number as well. This opens a new perspective in this area and calls for further research. With the advance of new and unused methods, we should reexamine old data about GM induced proliferation and apoptosis in kidney. Mesangial proliferation is mediated by stimulation of ROS and NO production (Martinez-Salgado et al., 2005[47]). GM stimulates expression of iNOS and NO production in isolated glomerulus and mesangial cells (Leung et al., 2004[41]). The increase in available NO during the course of oxidative stress can react with superoxide anion and peroxynitrite can be created. The created peroxynitrite is further creating nitrosative stress and cytotoxicity (Pedraza-Chaverri et al., 2004[60]). The precise role of balance between apoptosis and proliferation in mesangium is not clear so far. Probably one is a homeostatic consequence of the other. The primary effect *in vivo* would be apoptosis with following proliferation (Martinez-Salgado et al., 2007[46]). 

## Vascular Effects

The application of GM is followed by decrease in renal blood flow as a result of increased vascular resistance and not decreased perfusion pressure (Morales et al., 2002[52]). A drop in blood flow through the kidney result in decrease in glomerular filtration and available oxygen and ATP in tubules (Parsons et al., 1997[59]). In the beginning, the decrease in blood flow is a result of activation of tubuloglomerular feedback loop due to disturbance in tubular reabsorption, in order to prevent loss of fluid and electrolytes. Later on, after adaptation of this physiological mechanism, the flow is reduced because of vasoconstrictors production in renal blood vessels as well as by direct effect of GM on vascular cells. Nitric oxide produced in small quantities is important for physiological vasodilatation, while overproduction of NO due to overexpression of iNOS is causing cytotoxic effects. Nitric oxide is reacting with superoxide anion and peroxynitrite is formed, damaging proteins and cells. In such scenario, the formed peroxynitrite is turning endothelial NO synthase into dysfunctional enzyme which creates superoxide and contribute to vascular oxidative stress (Forstermann, 2010[23]). GM also interfere with relaxation of vascular smooth muscle cells, probably by stimulating vasoconstriction and reducing renal blood flow (Seçilmiş et al., 2005[79]). However, it was shown that GM can relax isolated precontracted arteries by inhibition of phospholipase C, protein kinase C and blocking of calcium channels (Wickman et al., 2001[92]). GM can induce leucocyte margination in retinal blood vessels leading to congestion and infarct. The observed effect is seen 48 to 72 hours after treatment (Hines et al., 1993[27]). It can be assumed that similar could happen in kidney, especially in proinflammatory environment. It is assumed that GM nephrotoxicity is primarily tubulopathy where tubular damage and dysfunction are main reasons for kidney failure. This can explain some clinical manifestations like proteinuria, enzymuria and electrolyte disbalance. However, in the absence of tubular obstruction, the sole tubular damage is insufficient for observed reduced glomerular filtration without additional extratubular events. Failure of tubular function leads to fluid and electrolytes loss which activates tubuloglomerular feedback loop, reducing blood flow and filtration. 

## Oxidative Stress

The treatment with GM produces oxidative stress in cells of the tubules, both *in vivo* and in culture (Karatas et al., 2004[35]; Juan et al., 2007[32]). The present oxidative stress is mediated by hydroxyl radicals from H_2_O_2_ and superoxide anion (Basnakian et al., 2002[4]). It is considered that oxidative stress has central role in GM induced nephrotoxicity (Abdel-Naim et al., 1999[2]). Such opinion is backed up with numerous experiments showing that cotreatment with different antioxidants protects from kidney damage induced by GM (Stojiljkovic et al., 2012[85]; Randjelovic et al., 2012[67][68]). However, some authors have shown that supplementation with glutathione (GSH) did not influence GM nephrotoxicity, regardless of decrease in lipid peroxidation and increase in level of GSH in kidney. GM directly increase production of ROS in mitochondria (Morales et al., 2010[53]). The created ROS inhibits respiratory chain and ATP production, stimulate release of cytochrome C and other proapoptotic factors, disturb cell function by damaging cellular proteins, lipids and nucleic acids, induce mesangial contraction, leads to endoplasmic reticulum stress, are involved in inflammation and inhibits transmembrane sodium transport causing cell swelling and necrosis. As stated earlier, produced NO by iNOS can react with superoxide anion and form peroxynitrite, highly reactive radical which damage the cell. Probably is the protective effect of antioxidants a result of joined action at different levels: attenuating cytotoxic effect of GM, inhibition of vasoconstriction and contraction of mesangium, anti-inflammatory effect. However, there is little information about the effect of antioxidants on cytotoxic effect of GM on cells of tubules in culture. Juan et al. (2007[32]) reported protective effect in similar conditions. They showed that tetrametilpirazin decrease accumulation of ROS and apoptosis in cells of rat kidney NFK-2E.

## Conclusion

Gentamicin induced nephrotoxicity has been extensively studied in the past and has become one of the established models of drug induced nephrotoxicity. However, there are still controversies and speculation about the precise mechanism of action. There are particularly unclear results about apoptosis and proliferation in glomeruli in gentamicin induced kidney damage. Here we tried to present current knowledge and some recent updates about balance between apoptosis and proliferation in glomeruli that shed new light into this model. This review can be used as a guide for everyone trying to understand and further investigate every aspect of gentamicin nephrotoxicity. Also potential readers could use presented ideas as a guide for their own future research.

## Acknowledgements

This work was supported by the Ministry of Science and Technological Development, Republic of Serbia (grants 43012 and 172061).
